# A Case of Lyme Disease Accompanied by Uveitis and White Dot Syndrome

**DOI:** 10.4274/tjo.25991

**Published:** 2016-10-17

**Authors:** İlkay Kılıç Müftüoğlu, Yonca Aydın Akova, Sirel Gür Güngör

**Affiliations:** 1 İstanbul Training and Research Hospital, Ophthalmology Clinic, İstanbul, Turkey; 2 Bayındır Kavaklıdere Hospital, Ophthalmology Clinic, Ankara, Turkey; 3 Başkent University Faculty of Medicine, Department of Ophthalmology, Ankara, Turkey

**Keywords:** Lyme disease, white dot syndromes, retinal vasculitis, Uveitis

## Abstract

In this case report we aimed to present a case of Lyme disease presenting as peripheral retinal vasculitis, intermediate uveitis and multifocal white dots in the posterior pole. The patient exhibited vitritis and snowball opacities in both eyes. A diagnosis of Lyme disease was made based on clinical, angiographic and laboratory findings. Fundus fluorescein angiography revealed optic nerve and retinal venous leakage as well as multiple hyperfluorescent foci in both eyes. The patient’s symptoms and ocular findings significant improved after treatment with a combination of systemic antibiotics and steroids. Ophthalmologists should bear in mind that conditions presenting with uveitis and multifocal white dots may be related to Lyme disease.

## INTRODUCTION

Lyme disease is the most common arthropod-related infectious disease caused by a spirochete known as Borrelia burgdorferi.^1^ Ocular involvement of Lyme disease is characterized by conjunctivitis, episcleritis, keratitis, uveitis, neuroretinitis, retinal vasculitis and cranial nerve palsies.^2^ It is probably underdiagnosed by ophthalmologists due to difficulty in the serologic diagnosis of the disease, as well as its nonspecific symptoms.

In this case report, we present a case of Lyme borreliosis and aim to point out a possible association between Lyme disease and white dot syndromes.

## CASE REPORT

A 30-year-old male patient with arthralgia had a history of an erythema on his leg resembling erythema nodosum. His best-corrected visual acuity was 20/20 in both eyes and intraocular pressure was normal at presentation. On slit-lamp examination, there was mild anterior chamber reaction and 2+ vitreous cells in both eyes. Fundus examination revealed snowball opacities and vascular sheathing in the inferior quadrants of the retina in both eyes (Figure 1a). Fluorescein angiography showed optic disc and retinal vascular leakage, and hyperfluorescent foci (Figure 1b, 1c, 1d). Indocyanine green angiography also showed hyperfluorescent foci at the posterior pole and in the peripheral fundus (Figure 1e, 1f, 1g, 1h).

**Figure 2a,b:**
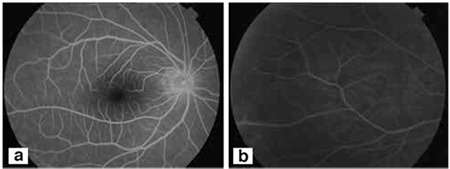
Fundus fluorescein angiogram of the patient following medical treatment

From the patient’s history we learned that he was working as a map engineer and had a history of trip to an endemic area. During his trip, he had noted erythema with papules on his leg, but had not assigned importance to the lesions, which spontaneously resolved. The patient was referred to the department of rheumatology for uveitis-associated systemic vasculitis work-up. The laboratory tests for sarcoidosis, syphilis and toxoplasmosis were within normal limits, except for elevated serum level of Lyme immunoglobulin M (350, normal range: 40-230) and positive immunoblot analysis. The patient did not exhibit any signs of Behçet’s disease. Given the findings and medical history, he was diagnosed with Lyme disease and a combination of topical prednisolone acetate therapy and intravenous ceftriaxone (10 days) was given, followed by a 4-week course of amoxicillin/clavulanate and oral corticosteroid therapy. After 3 months of therapy, no cells were observed in anterior chamber and vitreous, and leakage from retinal veins was reduced in fluorescein angiography at final visit (Figure 2). No recurrence was observed over a 1.5-year follow-up period.

## DISCUSSION

The clinical features and course of Lyme borreliosis in various systems are well described in the literature, but there has been little attention paid to its ocular involvement. Ocular presentations of Lyme disease include conjunctivitis, episcleritis, keratitis, uveitis, neuroretinitis, retinal vasculitis and cranial nerve palsies.^2,3^ In 1991, Smith et al.^4^ published the first report of retinal vasculitis in patients with seroreactivity to Lyme borreliosis. Their two patients’ ocular findings resolved with a combination of systemic antibiotic and corticosteroid therapy. Recently, Mikkila et al.^5^ reported the largest case series of 20 patients with ocular Lyme disease; eight patients had retinal vasculitis, while the other cases developed ocular adnexa inflammation and neuro-ophthalmological disorder in addition to branch central retinal vein occlusion. Two of these cases were accompanied by intermediate uveitis and one of two presented with multiple hypofluorescent foci at the level of the retinal pigment epithelium.^5^ Our patient also had intermediate uveitis and multiple foci around the posterior pole.

Ocular Lyme disease may affect either retinal arteries or veins to different degrees. Arterial involvement includes sheathing, cotton wool spots and obstruction, while venous involvement includes sheathing, retinal hemorrhage, edema and branch retinal vein occlusion.^6^ Retinal vasculitis may occur around the macula as well as peripheral retina associated with anterior and/or posterior segment ocular inflammation. In our patient, we observed leakage from retinal veins only, which extended from mid-periphery to the far-periphery of the fundus with anterior chamber reaction in addition to snowball opacities in the inferior peripheral area.

Serologic tests are often used for the diagnosis of Lyme disease. The recommended protocol includes a 2-test approach: Enzyme-linked immunosorbent assay (ELISA) and Western blot (WB).^7^ In this method, specimens are first tested by ELISA and then WB assay is used to confirm positive ELISA results. Because of limited sensitivity and specificity, these tests may be insufficient to diagnose current infection. Therefore the clinician should use both clinical findings and laboratory tests in order to diagnose Lyme disease, as in our case.

White dot syndromes have been associated with various diseases that are characterized by delayed hypersensitivity reaction such as sarcoidosis, tuberculosis, schistosomiasis and also Lyme disease.^8,9,10,11^ Despite these reports, in a study with 18 patients who showed all characteristic fundus and angiographic signs of white dot syndromes and had elevated serum levels of Borrelia burgdorferi-specific antibodies, no patient demonstrated evidence of Borrelia using immunoblotting methods.^11^

Although the exact pathogenesis in white dot syndromes is controversial, we thought that the relationship between these two conditions may be a result of the common pathology, vasculitis, which is also responsible for complications in the late phase of Lyme disease.

We treated our patient with systemic antibiotics in combination with oral and topical steroids. Although some manifestations of Lyme disease may resolve without antibiotic treatment; there is an increased risk of recurrence and progression to serious complications in the absence of antibiotic therapy.^12^ No recurrence was observed in our case. Therefore, the recommended treatment is antibiotics therapy combined with systemic steroids in severe cases.^6^

In our study, we report a patient with retinal vasculitis, snowball opacities and multifocal dots. Lyme disease, although rarely encountered, should be considered in the differential diagnosis of white dot syndromes.

### Ethics

Informed Consent: It was taken.

Peer-review: Externally and internally peer-reviewed.

## Figures and Tables

**Figure 1a,b,c,d:**
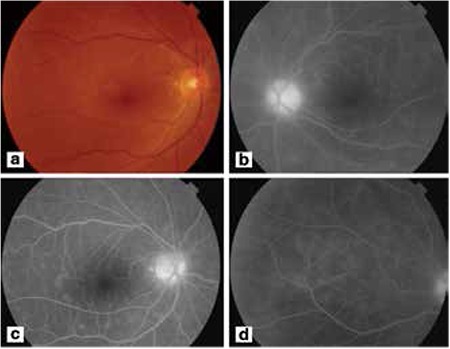
The fundus photograph and fluorescein angiography findings of the patient. Fundus photographs showed normal disc, macula and vascular archs (a), fluorescein angiography revealed leakage of the optic nerve heads, retinal veins and hyperfluorescence around the macula (b, c, d)

**Figure 1e,f,g,h:**
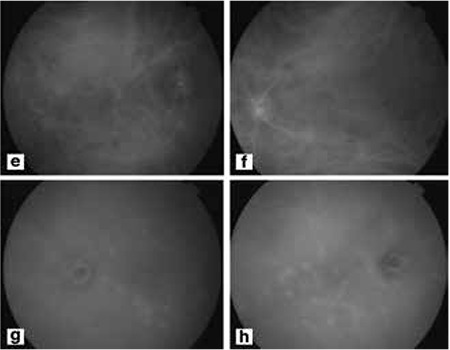
Indocyanine green angiography revealed hyperfluorescent focuses around the macula
